# Self-supervised learning of cell type specificity from immunohistochemical images

**DOI:** 10.1093/bioinformatics/btac263

**Published:** 2022-06-27

**Authors:** Michael Murphy, Stefanie Jegelka, Ernest Fraenkel

**Affiliations:** Department of Biological Engineering, Massachusetts Institute of Technology, Cambridge, MA, USA; Computer Science and Artificial Intelligence Laboratory, Massachusetts Institute of Technology, Cambridge, MA, USA; Computer Science and Artificial Intelligence Laboratory, Massachusetts Institute of Technology, Cambridge, MA, USA; Department of Biological Engineering, Massachusetts Institute of Technology, Cambridge, MA, USA

## Abstract

**Motivation:**

Advances in bioimaging now permit *in situ* proteomic characterization of cell–cell interactions in complex tissues, with important applications across a spectrum of biological problems from development to disease. These methods depend on selection of antibodies targeting proteins that are expressed specifically in particular cell types. Candidate marker proteins are often identified from single-cell transcriptomic data, with variable rates of success, in part due to divergence between expression levels of proteins and the genes that encode them. In principle, marker identification could be improved by using existing databases of immunohistochemistry for thousands of antibodies in human tissue, such as the Human Protein Atlas. However, these data lack detailed annotations of the types of cells in each image.

**Results:**

We develop a method to predict cell type specificity of protein markers from unlabeled images. We train a convolutional neural network with a self-supervised objective to generate embeddings of the images. Using non-linear dimensionality reduction, we observe that the model clusters images according to cell types and anatomical regions for which the stained proteins are specific. We then use estimates of cell type specificity derived from an independent single-cell transcriptomics dataset to train an image classifier, without requiring any human labelling of images. Our scheme demonstrates superior classification of known proteomic markers in kidney compared to selection via single-cell transcriptomics.

**Availability and implementation:**

Code and trained model are available at www.github.com/murphy17/HPA-SimCLR.

**Supplementary information:**

Supplementary data are available at *Bioinformatics* online.

## 1 Introduction

A number of technologies for multiplexed antibody-based tissue imaging have been developed in the past few years. These permit *in situ* characterization of cell-to-cell surface interactions and their intracellular proteomic correlates (Giesen *et al.*, 2014; Goltsev *et al.*, 2018; Gut *et al.*, 2018; Lin *et al.*, 2015; Schürch *et al.*, 2020), at high spatial resolution, via cyclic staining of the sample with antibodies conjugated to fluorophores (Lin *et al.*, 2015), nucleotide barcodes (Goltsev *et al.*, 2018) or metallic tags (Giesen *et al.*, 2014). A key consideration in such experiments is the selection of an antibody panel, which can be a difficult and lengthy process. A basic criterion for this panel is to incorporate antibodies for marker proteins specific to each cell type of interest in the tissue under investigation. While suitable antibodies are readily available for certain well-studied cell types (Laboratory of Systems Pharmacology, 2021), large-scale efforts such as the Human Cell Atlas (Regev *et al.*, 2017) promise to identify rarer cell types in the human body, for which selection of reliable antibody markers of cell type becomes an important and non-trivial consideration in experimental design.

Single-cell transcriptomics can be used to resolve the cell types comprising a tissue sample and their transcriptional profiles (Kiselev *et al.*, 2017). A number of recent efforts (Delaney *et al.*, 2019; Dumitrascu *et al.*, 2021; Qiu *et al.*, 2021) study the problem of identifying cell type-specific marker genes from transcriptomic data. However, direct application of such approaches to the proteomic context assumes the availability of antibodies targeting the proteins of the selected marker genes, and that such antibodies have been validated in the tissue of interest. This is a strong constraint: while the literature continues to grow, Lin *et al.* (2015) currently list only 257 antibodies demonstrated to work reliably with their approach (Laboratory of Systems Pharmacology, 2021).

Furthermore, even if a high-quality, validated antibody is available targeting a marker gene discovered from single-cell RNA sequencing data of a particular cell type of interest, if this gene is to be a useful marker *proteomically* in the tissue of interest, its transcript and protein levels also must strongly correlate in the tissue of interest. This is not universally the case, even for marker genes (Gong *et al.*, 2017; Stoeckius *et al.*, 2017; Zhou *et al.*, 2020).

Finally, biases in single-cell sequencing protocols can lead to undersampling of certain subpopulations of cells, which negatively affects the ability to detect marker genes via differential expression (Denisenko *et al.*, 2020; Ding *et al.*, 2020).

### 1.1 Human Protein Atlas

The Human Protein Atlas (HPA) is a large-scale compendium of proteomic experiments, dating back to 2003 and spanning several technologies including imaging, mass spectrometry and bulk RNA sequencing (Uhlén *et al.*, 2015). Among the datasets incorporated in the HPA is a large-scale immunohistochemistry (IHC) screen, in which tens of thousands of antibodies have been imaged in tissue microarrays representing several major organs in the human body. In the IHC protocol employed by HPA, a single antibody is imaged in each sample. The antibody stains brown wherever it binds a protein target, and a hematoxylin counterstain indicates nucleic acids in blue (Kampf *et al.*, 2012).

In principle, the HPA screen could also aid in selection of marker antibodies, if we could reliably determine the type of each imaged cell. However, the HPA screen measures only one antibody at a time. As a result, the staining pattern cannot be directly compared against canonical cell type markers in order to establish its cell type specificity. At present, resolving the staining pattern in an IHC image by cell type entails visual interpretation by a human expert (Uhlén *et al.*, 2015). This is prohibitive to carry out at finer spatial resolution than coarse anatomical regions for large numbers of images, and is necessarily subjective in nature. Although cell type labels are provided in the HPA, they are coarser than those discernible in modern single-cell sequencing experiments: for example, most images of kidney in the HPA are only annotated for staining in ‘glomeruli’ or ‘tubules’, while single-cell RNAseq experiments have defined, at varying resolution, between 13 (Muto *et al.*, 2021) and 100 (Lake *et al.*, 2021) types and subtypes of cells in the kidney.

### 1.2 Self-supervised learning

Learning informative representations of images without human supervision has been a long-standing problem in machine learning. One approach to this problem is self-supervised learning, which trains a classification or generative model to predict some attribute of the data that can be derived without a human labeler: for example, colorizing grayscale images (Zhang *et al.*, 2016), identifying distorted copies of an image (Dosovitskiy *et al.*, 2016; Gidaris *et al.*, 2018) or imputing masked patches (Pathak *et al.*, 2016). The representation of the data learned by the self-supervised model is then used as input to a simpler supervised learning model: very often, features extracted by the model as relevant for the self-supervised task should also be relevant for solving the supervised learning task.

Recently, self-supervised methods that employ *contrastive learning* have even outperformed supervised pre-training on large-scale image recognition tasks (He *et al.*, 2020; Misra and van der Maaten, 2020). The idea of contrastive learning is to learn a representation in which semantically similar (positive) pairs of observations $\left(x,x^{+}\right)$ are placed nearby, while semantically dissimilar (negative) pairs $\left(x,x^{-}\right)$ are placed far apart. This is achieved by learning an encoder *f* that minimizes the contrastive loss function (van den Oord *et al.*, 2018):

(1) $$E_{x,x^{+},{\left\{x_{i}^{-}\right\}}_{i=1}^{B}}-\text{log}\;\frac{e^{f{\left(x\right)}^{⊤}f(x^{+})}}{e^{f{\left(x\right)}^{⊤}f(x^{+})}+\sum_{i=1}^{B}e^{f{\left(x\right)}^{⊤}f(x_{i}^{-})}},$$

where typically a minibatch of *B* negative examples $x_{i}^{-}$ is used per query *x* instead of just one.

Since this approach does not use any human supervision, the semantic content of an image (e.g. its class label) is not available, and (dis)similarity information must be derived automatically. Contrastive learning generates positive examples for a given *x* via data augmentation that preserves semantics, e.g. randomly cropping, rotating or tinting. Negative examples are obtained by sampling the training set uniformly or by more sophisticated schemes (Robinson *et al.*, 2021; Chuang *et al.*, 2020).

### 1.3 Contributions

In short, this work makes the following contributions:

- We show how to effectively apply self-supervised learning to IHC images, by developing a sampling procedure that generates meaningful positive and negative examples, rather than having to engineer complex data augmentations;
- We show that the learned embeddings can be combined with labelled data from a different source—single-cell transcriptomics—to obtain a classifier of cell type specificity, without requiring human labelling of images; and
- We show applying both steps to IHC of kidney yields a representation that clusters images according to cell type specificity, and better classifies known proteomic markers than a purely transcriptomic approach.

### 1.4 Related work

Unsupervised and self-supervised learning have been employed previously to generate biologically informative representations of genes and proteins from high-throughput imaging data (Kobayashi *et al.*, 2021; Lu *et al.*, 2019; Pratapa *et al.*, 2021). However, the cited works focus on immunofluorescence imaging acquired in immortalized cell lines: while such datasets can indicate subcellular localization for thousands of proteins (Ouyang *et al.*, 2019), they are by nature uninformative of cell type and tissue specificity. Immunofluorescence images of single cells in culture also exhibit less sample-to-sample variability than IHC of tissue sections from human donors (Chatterjee, 2014; Im *et al.*, 2018; Tellez *et al.*, 2019). Here, we advance contrastive learning as a means of imposing invariance to sources of variability specific to IHC.

Supervised learning has been applied previously to the HPA IHC dataset, also for predicting subcellular localization (Hu *et al.*, 2022; Li *et al.*, 2012; Long *et al.*, 2020; Newberg and Murphy, 2008). Ghoshal *et al.* (2021) train a Bayesian neural network to classify cell type specificity of proteins imaged in IHC of testis, for which they rely on a training set of images manually annotated with cell type labels. In contrast, here we demonstrate how embeddings of IHC images learned via self-supervision can be combined with independent single-cell transcriptomics to predict cell type specificity without the need for human labeling beforehand.

Others have used deep learning representations to integrate imaging with transcriptomics data: Ash *et al.* (2021) use canonical correlation analysis of paired bulk RNAseq and autoencoder representations of H&E images to identify gene sets associated with morphological features, and Badea and Stănescu (2020) use intermediate activations of a classifier for the same problem. While our procedure also exploits correlation of morphology and gene expression, the problem we address in this article is fundamentally different: we seek to establish cell type specificities of proteins to facilitate antibody selection in experimental design, while the aforementioned are concerned with linking transcriptional programs and morphological phenotypes.

## 2 Materials and methods

### 2.1 HPA immunohistochemistry

The HPA includes approximately seven million IHC images spanning tens of thousands of antibodies, in tissue microarrays derived from tens of major tissues (Kampf *et al.*, 2012; Uhlén *et al.*, 2015). Each image represents a circular section of tissue between 0.6 mm-2mm in diameter that has been stained with an antibody and a hematoxylin counterstain. There are typically three replicated images per antibody, each derived from a tissue sample from a different donor. Each image is captured via microscope at 20× optical magnification, and provided via the HPA website at a resolution of approximately 3000 × 3000 pixels in medium-quality JPEG format. The gene whose product is nominally targeted by the antibody is indicated, as is its ‘staining’ classification: a qualitative assessment of the intensity of the antibody signal as ‘high’, ‘medium’, ‘low’ or ‘not detected’. An anonymized identification number for the donor of the tissue sample is also provided.

Each antibody in the HPA also undergoes a validation procedure to determine its binding specificity. Antibodies with ‘enhanced’ validation satisfy two criteria: (i) an antibody passes *independent antibody* validation if it displays the same staining pattern as another antibody targeting a non-overlapping epitope of the same protein in at least two tissues; (ii) an antibody passes *orthogonal* validation if its overall staining intensity matches expression of its nominal gene target in bulk RNASeq across at least two tissues. Both criteria are determined qualitatively by a human evaluator. In principle, it is unlikely for an antibody to satisfy both of these criteria yet bind to something other than its nominal target (Uhlen *et al.*, 2016).

In this work, due to storage and processing limitations we restrict our scope to IHC images from version 21 of the HPA taken in a single tissue: healthy kidney. Kidney was selected in particular because it is a complex organ that displays substantial anatomical and cellular diversity (Schumacher *et al.*, 2021) and with which the authors of this article are familiar. We further filter these to only include images of antibodies that passed ‘enhanced’ validation, that display either ‘medium’ or ‘high’ stain intensity, and that nominally target exactly one gene. This yields a training set comprising 10 164 images of immunostained kidney sections covering 2106 genes. Due to practical storage and memory limitations we also downsample these images to 512 × 512 pixels. This averages out finer-scale detail, but as we later demonstrate, still suffices to yield an informative embedding. We nonetheless anticipate our results will improve as image resolution is improved and the model is scaled accordingly.

### 2.2 Contrastively learning representations of immunohistochemical images

**Fig. 1.**
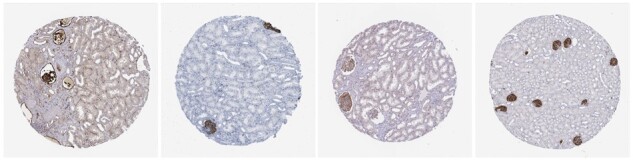
Semantically equivalent IHC images (here all of antibodies targeting the gene PODXL) display substantial variation in morphology and staining. Such artefacts would be difficult to capture via data augmentation

Contrastive learning is an effective approach for generating representations of images, both for visualization via dimensionality reduction and downstream classification tasks. This effectiveness depends on having meaningful data augmentations to generate positive examples. Simple image transformations that mimic natural variations in pose and lighting are sufficient for successful applications of contrastive learning to large datasets of natural images (Chen *et al.*, 2020a,b; He *et al.*, 2020). However, semantically equivalent biological images (e.g. IHC images of different markers of the same cell type) can display much more complex variability. Examples include morphological differences across tissue donors (due to e.g. age, sex, disease status), as well as technical artefacts (tearing or folding during tissue preparation, stain discoloration across batches)—all of which pose challenges for machine learning analysis of histological images generally (Komura and Ishikawa, 2018; Kothari *et al.*, 2014; Tellez *et al.*, 2019). Figure 1 provides an example of such variability among semantically equivalent images in the HPA. Augmentations capturing variability of the sort shown in Figure 1 would be difficult to engineer. Hence, instead of explicitly identifying and modeling such sources of variation, we couple the contrastive learning algorithm SimCLR (Chen *et al.*, 2020b) to a biologically informed scheme we develop for sampling positive and negative examples. We observe that the HPA already provides multiple semantically equivalent views at the level of *genes*: in addition to having multiple biologically replicated images per antibody of tissue samples from different donors, we also often have images of multiple antibodies that target the same gene. Therefore, rather than individual images, we treat the gene as the ‘observational unit’ in our data: for each gene, we sample a positive pair of images from the ‘equivalence class’ defined by all the images of antibodies for which that gene is the nominal target. To further increase the diversity of our training set, following this sampling procedure we also apply standard image augmentations to each member of the positive pair independently: specifically, we randomly crop each image to a 256 × 256 patch, then randomly apply HSV color jitter, scaling and rotation.

However, the image embeddings resulting from this approach displayed substantial clustering according to the donors from which they were derived. We believe this arises because (as we show in Supplementary Fig. S1) the donors do not co-occur uniformly across genes: the resulting imbalance in the positive pairs biases the encoder to push together images from donors that co-occur more frequently, giving rise to this (undesirable) clustering structure. We design the sampling of negative pairs to counteract this effect, noting that modified schemes for negative sampling are effective in contrastively learning debiased representations (Chuang *et al.*, 2020) and promoting inter-class separation (Robinson *et al.*, 2021). Here, rather than sampling uniformly, we only draw negative examples from the same donor: this steers the encoder to pull such clusters apart. We also provide further exposition and quantitative evaluation of the different sampling methods in Supplementary Appendix SA.

Rather than implementing a complicated scheme to generate stratified minibatches, we simply implement this by masking negative pairs from different donors within the minibatch when computing the contrastive loss. Defining the normalization term $B\prime =\sum_{i=1}^{B}1_{d=d_{i}}$ to be the number of negative examples sampled from the same donor *d* as image *x*, we use the following expression for the summand in Equation (1):

(2) $$-\text{log}\;\frac{e^{f{\left(x\right)}^{⊤}f(x^{+})}}{e^{f{\left(x\right)}^{⊤}f(x^{+})}+\left(B/B\prime \right)\sum_{i=1}^{B}1_{d=d_{i}}e^{f{\left(x\right)}^{⊤}f(x_{i}^{-})}}.$$

Algorithm 2.2 shows pseudocode of our entire sampling procedure.

**Algorithm 1** SimCLR with our modified sampling procedure. **Require:** batch size *B*, temperature *τ*, encoder network *f*, projection network ϕ, set of augmentations T, partition of dataset into genes $X_{g}$  **while** termination criterion is not met **do**

 sample minibatch of genes ${\left\{g_{i}\right\}}_{i=1}^{B}$

 **for all**  i∈1,…,B  **do**

  draw two images with donor labels $(x,d)∼X_{g_{i}},(x\prime ,d\prime )∼X_{g_{i}}$

  draw two augmentation functions t∼T,t′∼T

  $\tilde{x_{i}},\tilde{x}\prime_{i}=t(x_{i}),t\prime (x\prime_{i})$ # augmentation

  $h_{i},h\prime_{i}=f({\tilde{x}}_{i}),f(\tilde{x}\prime_{i})$ # representation

  $z_{i},z\prime_{i}=\phi (h_{i}),\phi (h\prime_{i})$ # projection

 **end** **for**

 **for all**  i∈1,…,B and j∈1,…,B  **do**

  $s_{i,j}=z_{i}^{T}z\prime_{j}/(||z_{i}||||z\prime_{j}||)$ # pairwise similarity

 **end for**

 **for all**  i∈1,…,B  **do**

  $B_{i}=\sum_{k=1}^{B}1_{d_{i}=d_{k}}$ # normalization factor

  $l_{i}=-\text{log}\;\frac{\;\text{exp}\;(s_{i,i}/\tau )}{\;\text{exp}\;(s_{i,i}/\tau )+(B/B_{i})\sum_{k\neq i}1_{d_{i}=d_{k}}\;\text{exp}\;(s_{i,k}/\tau )}$

 **end for**

 update networks *f* and ϕ to minimize $\ell =\frac{1}{B}\sum_{i=1}^{B}l_{i}$

**end while**

return encoder network *f*

### 2.3 Encoder architecture and training

As our encoder network *f* we use a DenseNet-121 (Huang *et al.*, 2017) convolutional neural network, which is initialized from weights pre-trained on ImageNet (Deng *et al.*, 2009). We use this architecture because it was selected by the top performer in the HPA subcellular localization challenge of Ouyang *et al.* (2019). We pass 256 × 256 RGB image patches into this encoder, which transforms them to an 8 × 8 grid of 1024-dimensional embeddings. We then average-pool this grid to a single 1024-dimensional vector, and linearly transform it to a *D *=* *128-dimensional real-valued embedding *h*. The projection head ϕ (Chen *et al.*, 2020b) then applies a ReLU non-linearity, followed by a second 128d linear transformation, and normalization to the unit *L*_2_-ball. This yields a vector *z* on which we compute the contrastive loss. We use a temperature parameter of τ=1.0.

The model is implemented in PyTorch Lightning (Paszke *et al.*, 2019) using the Kornia (Riba *et al.*, 2020) library for data augmentation. We fit this model using the Adam optimizer (Kingma and Ba, 2015) for 1000 epochs, with learning rate $5\times 10^{-4}$ and batches of size 150, using 4 nVidia Volta V100 GPUs with 32GB VRAM. Larger batch sizes did not fit in memory, even with the aid of PyTorch Lightning’s automatic half-precision casting, which we employ here. To avoid arithmetic underflow, we compute the contrastive loss at full 32-bit precision.

### 2.4 Learning cell type specificity from auxiliary transcriptomic labels

We next demonstrate how our embeddings can be used to classify IHC images according to cell type specificity of the stained protein. As we generally lack human labels of cell type specificity for these images, we instead train a classifier using auxiliary labels of specificity, derived from an independent single-cell transcriptomics dataset. Because transcription imperfectly correlates with protein expression, these labels act only as a noisy proxy of proteomic specificity. Therefore, we should not seek a complex model that achieves very low error—as this would likely capture noise that is unrelated to protein expression. Rather, by restricting to only simple functions of embedded protein images, we prevent the classifier from learning such noise, and steer it toward what we actually desire: predictions of proteomic specificities. In this work we select a linear classifier for this task.

As our single-cell transcriptomics dataset, we use the processed data provided by Muto *et al.* (2021) via the *cellxgene* portal (Chan-Zuckerberg Biohub, 2022). This dataset consists of single nuclei from non-tumor kidney cortex from five donors (three male, two female) sequenced using the 10× Genomics Chromium v2 platform. This yielded a matrix of 19 985 cells × 22 127 genes. Muto *et al.* (2021) performed unsupervised clustering of this data using the Louvain community-detection algorithm, and assigned a cell type to each cluster based on expression of known lineage-specific markers, resulting in 13 different cell types. The dataset provided on *cellxgene* also maps the shorthand names used by Muto *et al.* (2021) for these cell types to terms in the Cell Ontology (Meehan *et al.*, 2011), which we use in this article.

The learning problem is as follows: we first compute, for each gene *g*, its mean transcriptional expression in each of the *K* cell types annotated in the single-cell dataset. We then normalize this to a vector $y_{g}\in \Delta_{K}$, and train a linear classifier to predict *y_g_* from its corresponding embedded images $\left\{h_{i}\in {\mathbb{R}}^{D},i\in 1\ldots N:g_{i}=g\right\}$:

(3) $$\min_{A\in {\mathbb{R}}^{D\times K}}-\sum_{i=1}^{N}y_{g_{i}}^{⊤}\;\text{log}\;\left(\text{softmax}(h_{i}^{⊤}A)\right),$$

where log ⁡ is applied element-wise.

We fit the model in Equation (3) to the same training set as before, minus 633 images corresponding to a test set of marker genes indicated in (Habuka *et al.*, 2014). We train for 1000 epochs using the Adam optimizer with learning rate 0.01.

### 2.5 Evaluating predictions of proteomic specificity

As we lack large-scale annotation of cell type specificity for proteins in kidney, we instead assess our model’s predictions at the scale of coarser anatomical regions, and compare these to estimates of regional specificity derived solely from single-cell transcriptomic data.

We employ a list of marker genes identified as proteomically specific to particular kidney anatomical regions by Habuka *et al.* (2014). These markers were identified first by preselecting genes with kidney-specific expression in bulk RNAseq, and then labelled with one of four anatomical regions of kidney cortex by manual inspection of IHC images from an earlier version of the HPA. Of these markers, 126 are present both in version 21 of the HPA and in the single-cell transcriptomics dataset, and we limit our evaluation to these. We hold out the corresponding images of these genes during training of the classifier.

**Fig. 2.**
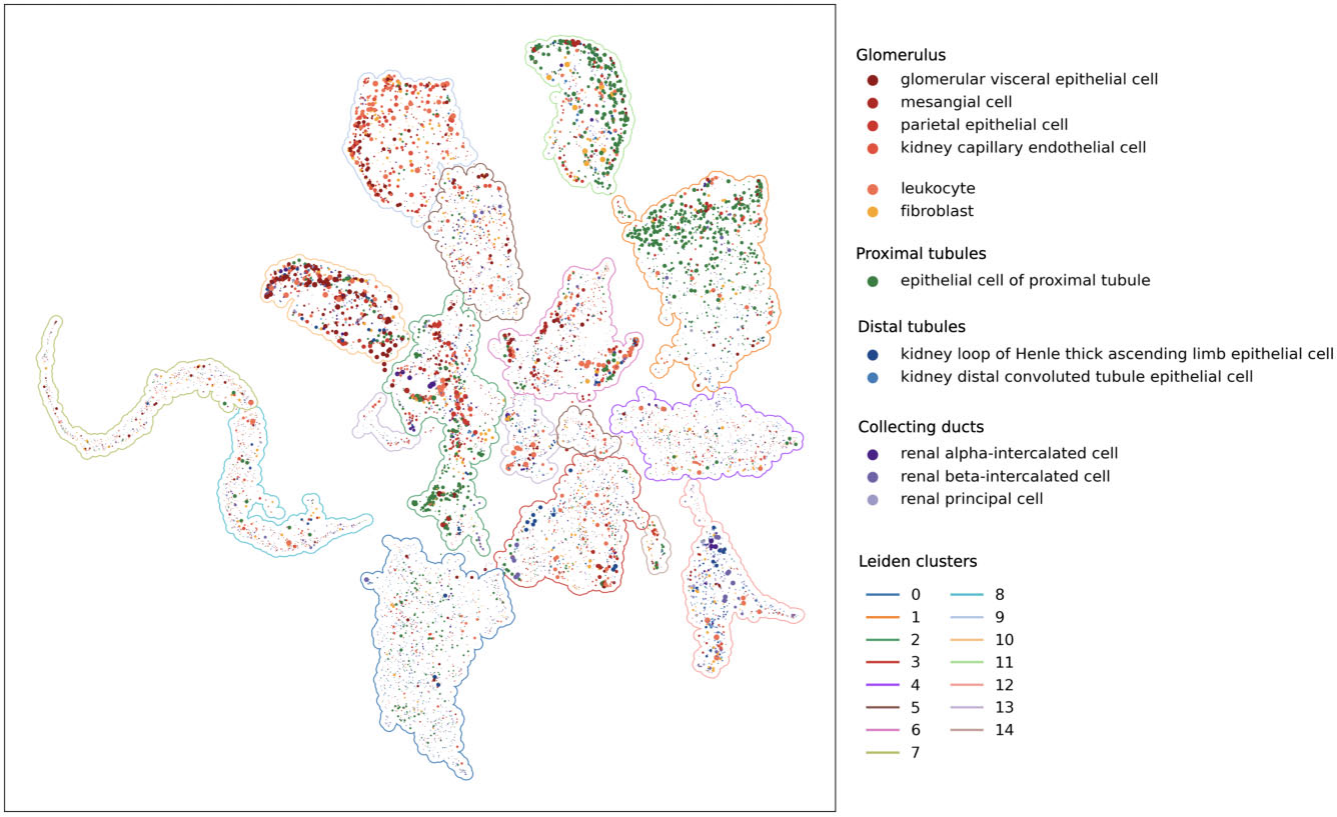
Visualization of 10 164 image embeddings via UMAP dimensionality reduction demonstrates that our method captures biological semantics of IHC images. Each dot represents an embedding of a single IHC image. We color the embedding according to the cell type in which its corresponding gene is most expressed, per the scRNA dataset of Muto *et al.* (2021), and size each according to cell type specificity (i.e. $\arg\max_{k}y_{gk}$ and $\max_{k}y_{gk}$ respectively). Cell types are grouped according to anatomical regions in Habuka *et al.* (2014); ‘leukocyte’ and ‘fibroblast’ do not correspond to any single region. Leiden clusters are numbered and outlined

The anatomical regions labelled in Habuka *et al.* (2014) each (with the exception of proximal tubules) contain multiple transcriptionally defined cell types. As our model is trained to predict cell types, we map cell types in Muto *et al.* (2021) to anatomical regions in Habuka *et al.* (2014) according to the scheme shown in Figure 2, omitting two cell types (‘leukocyte’ and ‘fibroblast’) from our analysis that lack a clear correspondence to a single anatomical region. We convert our model’s predictions from cell types to regions by summing the softmax probabilities for all cell types mapped to a given region, and then renormalizing. This yields, for each image, a vector of predicted probabilities for each of the four regions. But as our ground-truth labeling is only provided for genes (not for individual images), we generate predictions at the gene level by taking the average of all such vectors for a given gene.

As baselines, we consider assigning genes to cell types using the single-cell transcriptomics data alone. For each gene, we perform differential expression testing for each cell type, and use the test statistic as a score of cell type specificity. We then convert these scores per cell type into anatomical regions by taking the maximum test statistic over all cell types mapped to a given region. We do this separately for one-versus-rest T-tests and Wilcoxon tests [using ScanPy’s (Wolf *et al.*, 2018) sc.tl.rank_genes_groups function], which are common practice for marker detection in scRNA analysis (Luecken and Theis, 2019).

We also compare against a published state-of-the-art algorithm for marker detection, COMET (Delaney *et al.*, 2019). We run COMET using default parameters and consider only test statistics of positive singleton markers in our evaluation [as Habuka *et al.* (2014) only specify labels for these].

**Fig. 3.**
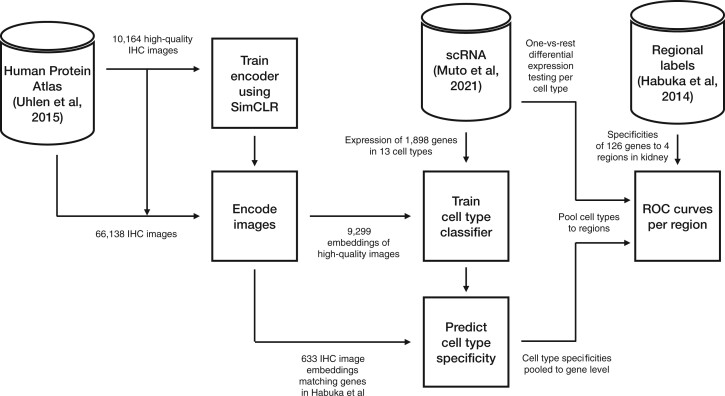
We first train an encoder on 10 164 IHC images from the HPA that pass quality filtering, and compute embeddings for all 66 138 images, which we subsequently hold fixed. We then compute mean expression in each of 13 cell types for each gene in Muto *et al.* (2021). We fit a linear classifier to embeddings of 9299 high-quality images that (i) correspond to 11 657 genes in Muto *et al.*, and (ii) are not listed in Habuka *et al.* (2014). We then evaluate this linear classifier on embeddings of 633 held-out HPA images matching 126 genes annotated as regional markers in Habuka *et al.* (2014). We aggregate along the image axis to yield gene-level predictions, and along the cell-type axis to yield predictions for four anatomical regions. This permits us to compute ROC curves using the labels of Habuka *et al.* (2014) and evaluate against baselines that rely upon differential expression of transcripts between cell types to nominate markers

We provide a schematic in Figure 3 that summarizes our training and evaluation workflow.

## 3 Results

### 3.1 Contrastively learned embedding structure reflects cell type specificity of immunohistochemical stains

We first use ScanPy’s UMAP dimensionality reduction (McInnes and Healy, 2018) to visualize the embeddings learned by our model. We also cluster these embeddings using Scanpy’s implementation of the Leiden community-detection algorithm (resolution parameter 0.2). This is shown in Figure 2, where we also color and size each image embedding according to the cell type in which the corresponding gene is most expressed, per scRNA from Muto *et al.* (2021). This reveals a number of clusters exhibiting specificity for particular cell types and anatomical regions in the kidney. Importantly, this structure arises solely from our self-supervised objective and without human supervision: at no point is cell type specificity of an image ever provided as a label to our algorithm.

**Fig. 4.**
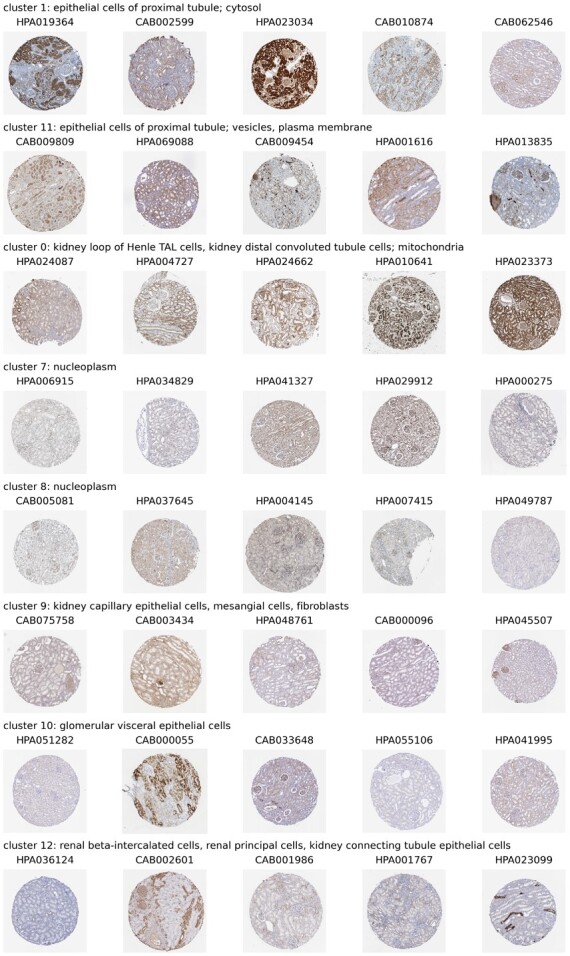
Exemplar images chosen from a subset of Leiden clusters with clear specificity for kidney anatomical regions and subcellular localizations, with HPA antibody codes

Exemplars from these clusters are shown in Figure 4. We identify these exemplars by training an SVM [scikit-learn (Pedregosa *et al.*, 2011), RBF kernel, default parameters, fivefold CV] to predict each embedding’s cluster label, extracting the top five highest-scoring images for each class.

Clusters 1 and 11 are enriched in genes displaying ‘epithelial cell of proximal tubule’-specific expression (hypergeometric test, $P<10^{-202}$ and $P<10^{-50}$ respectively).

While the reason for images of this cell type separating into two different clusters is unclear to the unaided eye, hypergeometric testing for subcellular localization annotations of the respective genes (Thul *et al.*, 2017) indicates cluster 1 primarily enriches for localization in the cytosol ($P<10^{-44}$), while cluster 11 enriches for both vesicles ($P<10^{-7}$) and plasma membrane ($P<10^{-8}$).

This suggests our self-supervised approach may also be sensitive to subcellular localization, confirming findings from the supervised setting (Hu *et al.*, 2022). Indeed, the images in cluster 0, which enrich for loop of Henle ($P<10^{-21}$) and distal tubule ($P<10^{-14}$) cells, are also strongly enriched for mitochondrial localization ($P<10^{-300}$). Similarly, clusters 7 and 8 enrich for nucleoplasmic localization ($P<10^{-170}$ and $P<10^{-152}$).

Clusters 9 and 10 both consist of genes expressed in the glomerulus. However, they display specificities for different cell types within that region: cluster 9 enriches for ‘kidney capillary epithelial cells’ ($P<10^{-31}$), ‘mesangial cells’ ($P<10^{-11}$) and ‘fibroblasts’ **(**$P<10^{-13})$, while cluster 10 enriches for ‘glomerular visceral epithelial cells’ ($P<10^{-30}$).

Another cluster with interpretable anatomical specificity is 12, which enriches for genes specific to ‘renal beta-intercalated cells’ ($P<10^{-16}$) and ‘renal principal cells’ ($P<10^{-18}$), both of which are found in the collecting duct of the kidney. The cluster is also enriched for ‘kidney connecting tubule epithelial cells’ ($P<10^{-22}$), which physically link the distal tubule to the connecting duct (Kitching and Hutton, 2016).

### 3.2 Biologically informed sampling captures semantic content and imparts invariance to donor identity

**Fig. 5.**
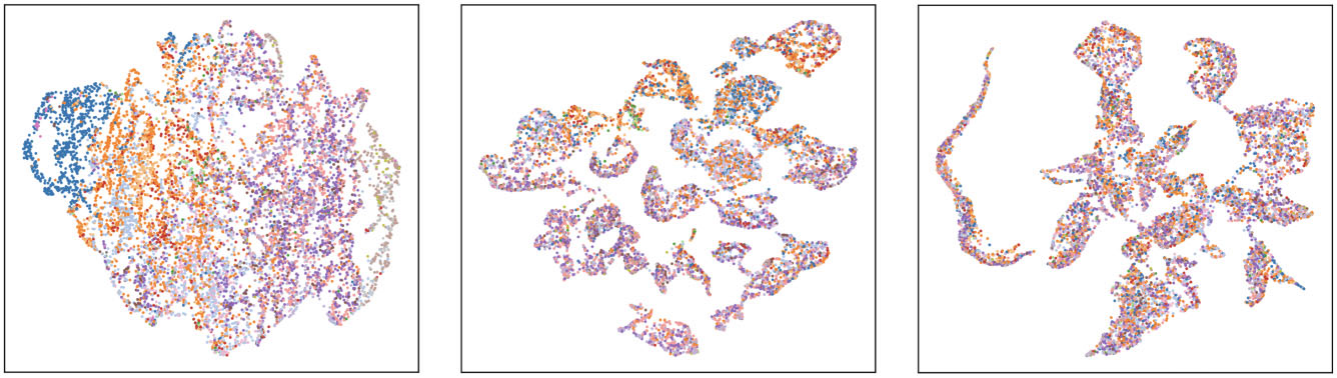
UMAP plots of image embeddings learned from three sampling schemes: image augmentations only (left); + grouping positive examples by gene (middle); + stratifying negatives by donor (right). Color indicates one of 18 anonymized donor labels. The last scheme leads to visibly better mixing of donors across clusters

Figure 5 displays the effect of our positive and negative sampling procedures via UMAP visualizations of the resulting embeddings.

We first consider a self-supervised encoder trained using only standard image augmentations (left). This does not result in a visualization with clear clustering structure, and images tend to colocate with other images derived from the same donor. In comparison, our approach of sampling positive examples from other images of the same gene (middle) imparts clearly visible global clustering structure.

However, there remain some clusters exhibiting visible imbalance with respect to the donor label. This is fixed when we additionally restrict negative examples to images derived from the same donor (right). We quantify invariance of our representation to the identity of the tissue donor via fivefold cross validation accuracy of a logistic regression trained to predict the donor label from the embedding. When sampling negatives uniformly, the donor label can be accurately classified 39.7% of the time; our negative sampling procedure makes this task more difficult, reducing to 29.5% accuracy.

### 3.3 Immunohistochemical classification yields superior predictions of regional specificity over transcriptomics

**Fig. 6.**
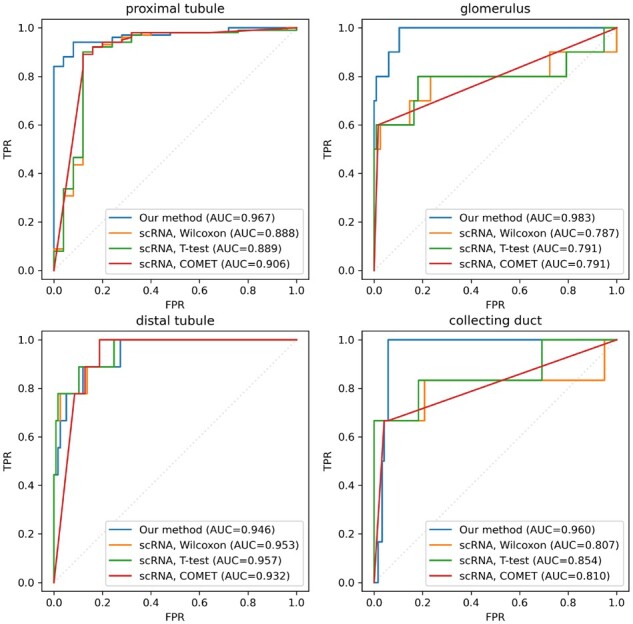
One-versus-rest ROC curves with respect to the labels of regional specificity in Habuka *et al.* (2014) for our classifier and the transcriptomic baselines

We evaluate our model’s performance in classifying images of proteomic markers for the four anatomical regions specified in Habuka *et al.* (2014), against the common practice of one-versus-rest *T*-tests and Wilcoxon tests (Luecken and Theis, 2019) as well as the state-of-the-art method COMET (Delaney *et al.*, 2019). Figure 6 shows one-versus-rest receiver operator characteristic curves for each region.

Our approach incorporating IHC demonstrates superior accuracy in proximal tubule (ΔAUC =0.061), glomerulus (ΔAUC =0.191), collecting duct (ΔAUC =0.106) and comparable accuracy in distal tubules (ΔAUC =−0.011) over the best transcriptional baseline for each region.

**Fig. 7.**
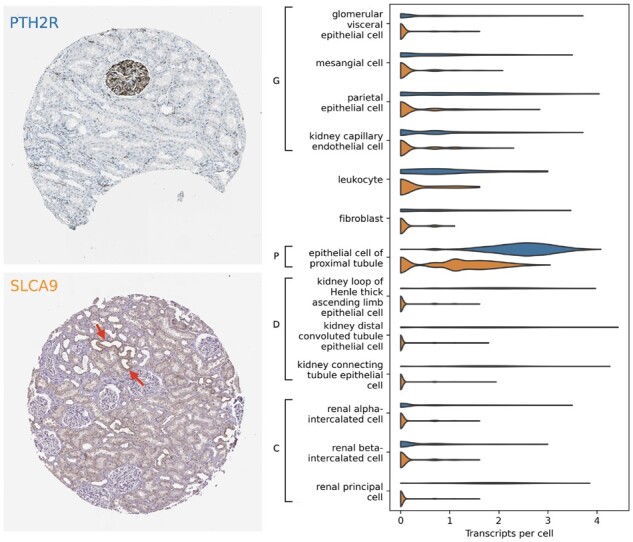
Example images of PTH2R (top; antibody HPA010655) and SLC2A9 (bottom; antibody HPA066229). Regions in the latter that appear to be stained distal tubules are indicated by red arrows. Violin plot indicates the transcriptional expression of PTH2R (blue) and SLC2A9 (orange) in each cell type of Muto *et al.* (2021). Brackets indicate anatomical regions (G, glomerulus; P, proximal tubule; D, distal tubule; C, collecting duct)

We provide two examples of markers for which immunohistochemical and transcriptomic predictions disagree. Figure 7 shows a selected image from each, along with the corresponding gene expression per each cell type in Muto *et al.* (2021).

Habuka *et al.* (2014) label PTH2R as a proteomic marker of cells in the glomerulus. Our model agrees, ranking the antibody HPA010655 for PTH2R third-most-specific to glomeruli of the 217 antibodies targeting genes in Habuka *et al.* (2014). In comparison, the overall best-performing scRNA baseline (*T*-test) ranks PTH2R *last* among the 126 marker genes by glomerular specificity, instead assigning it to proximal tubules (ranked 8th of 126).

We suggest an explanation for the misclassification based on scRNA: using *in situ* hybridization, Usdin *et al.* (1996) observe PTH2R is specifically transcribed in a small subpopulation of glomerular cells in rat kidney. It is possible that biases in the scRNA sequencing or analysis protocols negatively affected detection of that minority cell type (Denisenko *et al.*, 2020; Ding *et al.*, 2020). This would present a potential mode of failure when relying upon single-cell transcriptomics data to determine proteomic specificity.

Habuka *et al.* (2014) label SLC2A9 as a marker of proximal tubules. The best transcriptomic baseline accurately classifies this gene, ranking it 6th of 126 in proximal tubule specificity. Conversely, our method incorrectly classifies this gene’s antibody, HPA066229, as most specific to distal tubules (ranked 25th of 217, versus 187th for proximal tubules).

Rather than a mistake of our classifier, we suggest this may be a limitation of relying on IHC: visual inspection of the images of that gene also indicates staining of distal tubules, highlighted with red arrows in Figure 7. However, So and Thorens (2010) confirm Habuka *et al.* (2014)’s annotation, indicating SLC2A9 is known to selectively express in proximal tubules. This may indicate off-target binding of the antibody: while Habuka *et al.* (2014) may have observed and accounted for this in their annotation, our approach cannot discern such cases.

We also provide in Supplementary Appendix SB a demonstration of our method applied to IHC images and scRNA of testis. We benchmark against transcriptomic baselines as shown here, as well as DeepHistoClass (Ghoshal *et al.*, 2021), a *supervised* learning algorithm trained on images of testis that were manually labelled for cell-type specificity by human experts.

## 4 Discussion

In this article, we develop a contrastive learning algorithm for learning representations of IHC images in the HPA. We demonstrate our approach to sampling positive and negative examples leads to a representation that captures biological semantics of IHC images, without needing to engineer complex data augmentations. When applied to images of the kidney, the resulting embeddings yield clusters that display specificity for different cell types and anatomical regions. We then incorporate auxiliary labels from an independent single-cell transcriptomics dataset to train an image classifier that predicts cell type specificity without requiring human annotation of those cell types. This classifier better recovers known proteomic markers than prioritization solely via differential transcriptional expression.

One important potential application of our method is toward designing multiplexed spatial proteomics experiments, for which antibody panel selection is an important consideration. The ability to screen candidate marker genes beforehand for proteomic specificity, as opposed to solely transcriptomic specificity, should save time during validation of such panels.

While we only consider images of kidney here, our contrastive learning procedure is applicable to any tissue represented in the HPA, and the embeddings can be used for any prediction or visualization task for which invariances of the sort we learn are desirable. We also point out that while training the model does necessitate the unique scale of the HPA specifically, in principle it can be subsequently applied to any IHC image acquired in a tissue that was represented in the training set. Our subsequent classification step can also use any data type that associates genes or proteins with cell type specificity: it is not limited to our particular choice of single-cell transcriptomics dataset, nor to the granularity of cell type definitions therein. It therefore will benefit from efforts to discover and catalog finer distinctions between cell types, such as the Human Cell Atlas (Regev *et al.*, 2017).

In addition to cell type specificity, we also observe our approach is sensitive to subcellular localization of markers. This result is unsurprising, as multiple previous works demonstrate effective prediction of subcellular localization from IHC images in the supervised context (Hu *et al.*, 2022; Li *et al.*, 2012; Long *et al.*, 2020; Newberg and Murphy, 2008): here we find such information can also be detected without supervision. A representation that disentangles these two modes of specificity would be a promising direction of future work.

We note some limitations with our method in its current implementation. We had to downsample the high-resolution images in the HPA substantially to accommodate our choice of architecture, and GPU memory constraints prevented us from using larger batch sizes as recommended for SimCLR (Chen *et al.*, 2020b). We expect improvements in memory-efficient contrastive learning such as He *et al.* (2020) will permit us to use HPA images at their native resolution, which should yield finer distinctions between cell types and subcellular compartments. We also anticipate multi-scale attention (Tao *et al.*, 2020) will be particularly useful for distinguishing cell type markers by finer-scale features.

Finally, our approach is fundamentally limited by antibody cross-reactivity: while we filter our training set using the HPA’s reliability criteria to counteract this, in principle an approach that attempts to predict cell type specificity of proteins from immunohistochemical images will fail when antibodies bind to proteins other than their nominal targets. On the other hand, genes that exhibit systematic disagreement between immunohistochemical and transcriptomic estimates of cell type specificity across multiple antibodies and tissues may present interesting candidates for biological follow-up in their own right.

## Supplementary Material

btac263_Supplementary_Data
