# Supplementary material for: Self-supervised learning of cell type specificity from immunohistochemical images
Source: Bioinformatics. 2022 Jun 27;38(Suppl 1):i395–403. doi: 10.1093/bioinformatics/btac263 (PMC9235491; doi:10.1093/bioinformatics/btac263)
Supplement: btac263_Supplementary_Data [file btac263_supplementary_data.pdf]

Appendix A. Comparison of sampling procedures

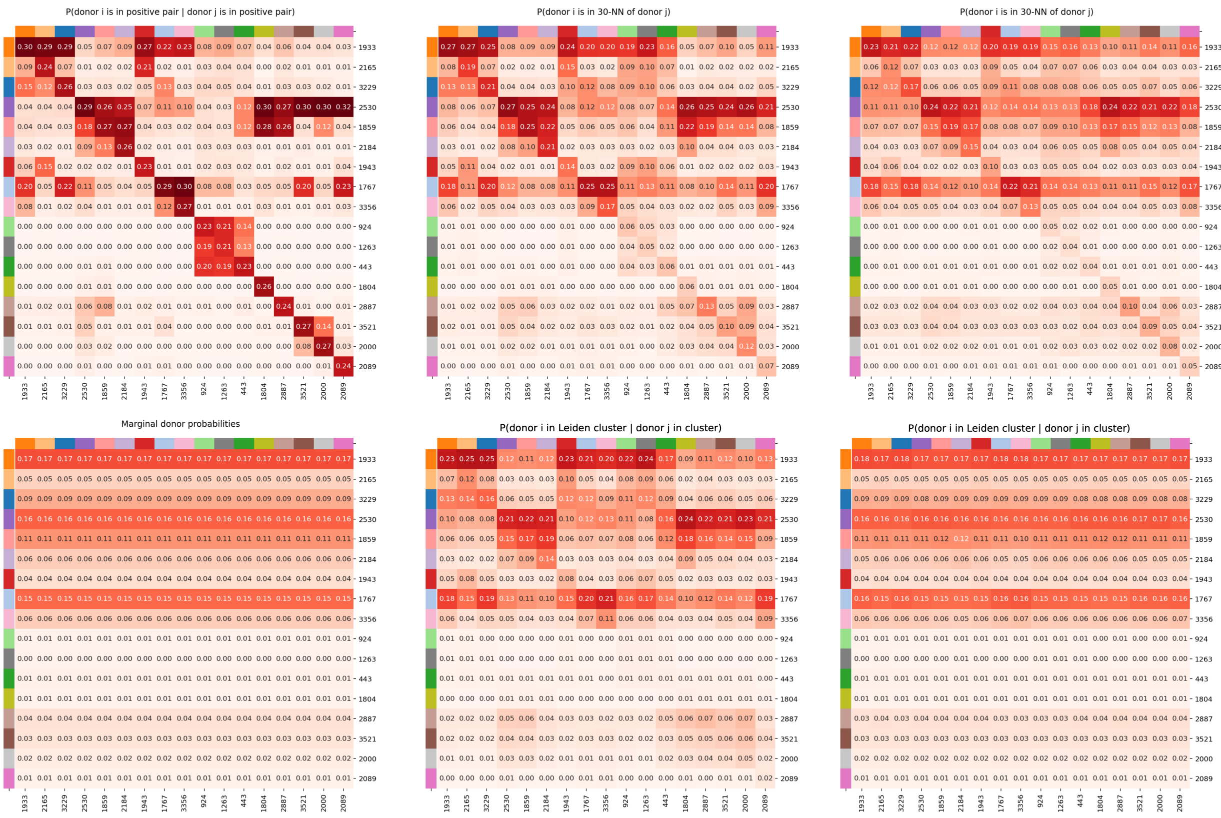

Figure S1: Top left, conditional probability of drawing a positive example from donor i for an image from donor j; top middle, conditional probability of an embedding of an image from donor i lying in the 30-nearest-neighbors of an embedding from donor j without our negative sampling procedure; top right, the same, with our negative sampling procedure; bottom left; marginal probability of drawing an example from donor i; bottom middle and bottom right, same as top but with Leiden clusters.

Figure S1 suggests the co-occurrence of donors among positive pairs is reflected in the geometry of the learned embeddings: specifically, the conditional probabilities of observing donor i as a positive example for donor j when grouping images by genes (top left), resemble the proportion of images from donor i in the 30 Euclidean nearest neighbors of an embedding of donor j (top middle; KL divergence between these two distributions for fixed j, averaged across all $j = 0.215$). The negative sampling procedure drives these two donor-conditional probability distributions farther apart (top right; average KL divergence $= 0.349$). It also drives the proportion of images of donor i in a 30-NN of donor j much closer to what would arise if each donor were just represented in the neighborhood proportionally to its total number of images (bottom left; average KL divergence 0.228 before, 0.082 after).

We can similarly compute the probability of observing an image from donor i within a Leiden cluster (resolution $= 0.2$) of the embeddings, conditional upon donor j also being present in that cluster. While the relation to the donor co-occurrence is weaker to begin with (bottom middle), the effect of the negative sampling is even stronger at the cluster level: donors are assigned to clusters essentially randomly (bottm right).

We further note it is not possible with this dataset to directly correct the imbalance in donor pairings by instead reweighting each gene's positives: generally only a few (median 3, maximum 6) of the 18 donors are represented per gene, so most pairings of donors will occur for that gene with probability zero.

In Table S1 we provide quantitative metrics for the effectiveness of the different strategies for sampling positive and negative pairs: both in terms of test performance on the downstream classification task, as

**Sampling method**	**AUC, P**	**AUC, G**	**AUC, D**	**AUC, C**	**AUC, overall**	**Accuracy, donor**
Image augmentation	0.957	0.941	0.798	0.883	0.895	0.621
Image augmentation + sampling at gene level	0.972	0.954	0.877	0.950	0.939	0.397
Image augmentation + sampling at gene level + rejecting positives	**0.989**	**0.991**	0.934	0.953	**0.967**	0.464
Image augmentation + sampling at gene level + rejecting negatives	0.967	0.982	**0.949**	**0.960**	0.964	**0.295**

Table S1: Performance of different sampling strategies on the downstream classification task (test AUC on proximal tubule (P), glomerulus (G), distal tubule (D), collecting duct (C), and overall; higher better), and invariance to donor identity of the embeddings (5-fold CV accuracy; lower better).

well as invariance of the embedding to the donor identity. As we describe in the main text, the latter is quantified via 5-fold cross validation accuracy of a logistic regression trained to predict the donor label from the embedding: a more invariant embedding will make this prediction task harder.

We also considered an alternative approach for sampling, which we include here for completeness: rather than only sampling negative pairs originating from the same donor, we only drew positive pairs from different donors. This was similarly implemented via masking at the minibatch level. This did not affect the classification accuracy of the downstream task (mean AUC = 0.967 across the four regions, versus 0.964 for negative sampling). It also achieved the opposite of our desired effect, *increasing* linear separability of donors. The embeddings derived from this approach, as well as those of the other three we investigated, are shown in Figure S2.

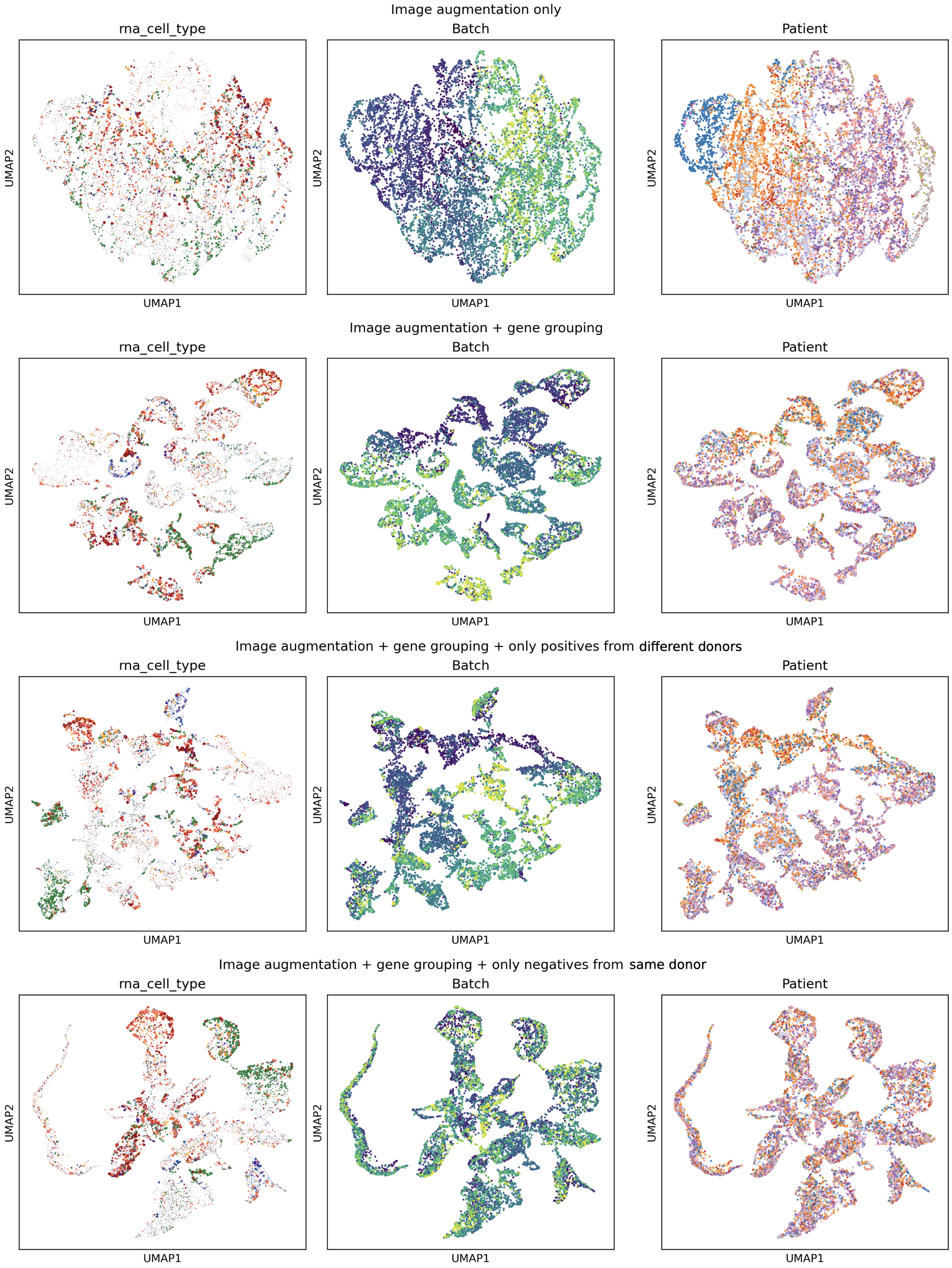

Figure S2: UMAP plots of IHC image embeddings from the four sampling schemes. The left column indicates the cell-type specificity of the respective gene in Muto *et al.* (2021). The middle column, "Batch", is an ordinal index of the individual TMA on which the image was acquired (each TMA contains three images of the same antibody, each from a different donor). It is clearly confounded with the donor label; despite only using the latter, our final scheme visibly achieves the best mixing across clusters of this label as well.

Appendix B. Evaluation on immunohistochemistry of testis

We also apply our method to a different tissue: healthy human testis. We select this tissue because (Ghoshal *et al.*, 2021) provide (1) a manually-annotated test set of IHC images with cell-type labels (as opposed to regional specificity), and (2) a supervised learning model, DeepHistoClass, that provides a proteomic benchmark against which we can evaluate our method.

To train our encoder, we use 4777 images of testis from version 21 of the Human Protein Atlas, selected using the same filtering criteria described in the main text for kidney. The model and training parameters are identical to the procedure described in the main text.

To train our classifier, we employ cell type specificities from the scRNA dataset of (Guo *et al.*, 2018). As the per-cell labels of cell type were not provided in the published data by the authors, we derive them by running ScanPy's Leiden clustering (default parameters) after log-transformation and PCA, and assign clusters to cell types via manual inspection of transcriptional marker genes described in (Guo *et al.*, 2018). UMAP plots labelled with these clusters are shown in Figure S3 and our assignments of these to the cell types in (Ghoshal *et al.*, 2021).

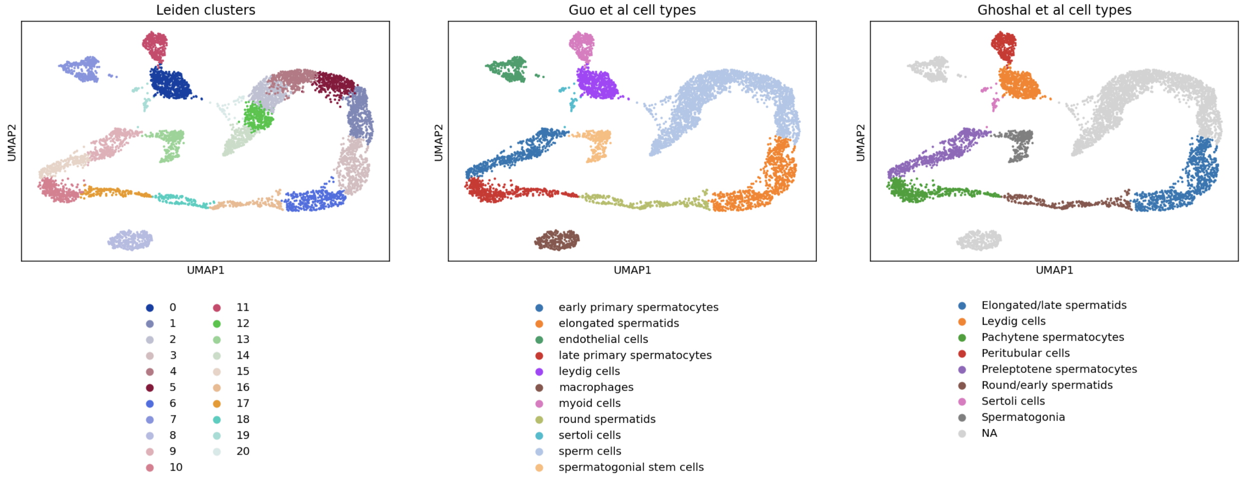

Figure S3: Labelled UMAP embeddings of the scRNA data in (Guo *et al.*, 2018). Each point represents a single cell. Cells labelled “NA” are not present among the labels in (Ghoshal *et al.*, 2021).

(Ghoshal *et al.*, 2021) provides a test set of human annotations for 1374 images from versions 18 and 19 of the Human Protein Atlas, which we also employ as a test set (after removing 31 images also present in our training set). Figure S4 shows the performance of their model, ours, and transcriptomic tests of differential expression using (Guo *et al.*, 2018). (For both image-based methods, as in the main text, we aggregate labels to gene-level via averaging over all images annotated with a given gene.)

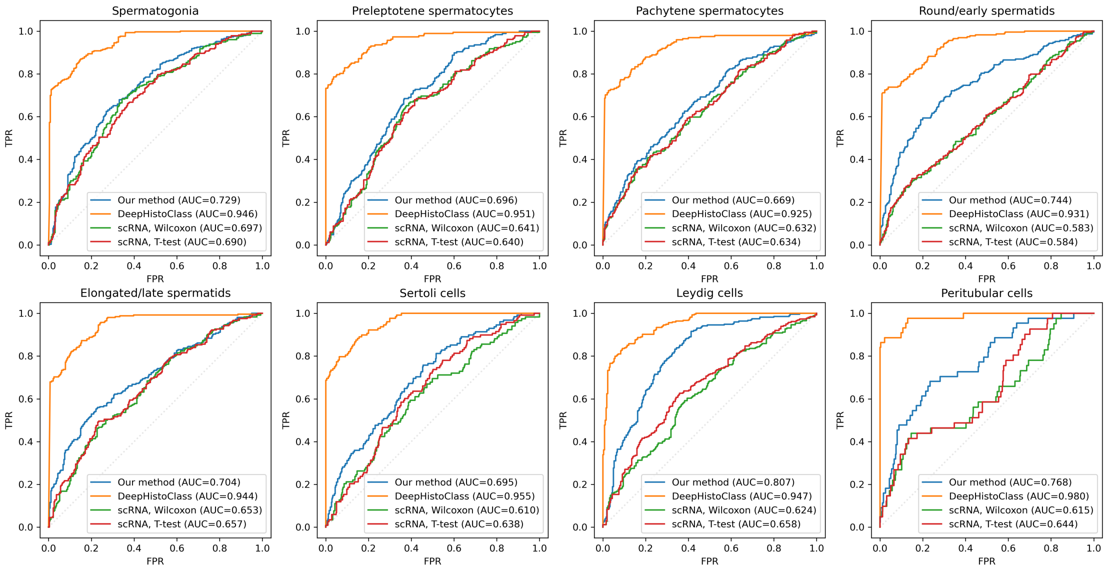

Figure S4: AUC curves of our method, DeepHistoClass, and two transcriptomic baselines for classifying marker genes in testis.

While we outperform the scRNA-only baselines, we observe that DeepHistoClass outperforms our approach in turn by a substantial margin. This is unsurprising. DeepHistoClass is a *supervised* learning algorithm, and therefore has access to direct labels of proteomic specificity at the level of individual images during training. By comparison, our method only has access to a noisy proxy in the form of transcriptomic specificity. The additional supervision information used by DeepHistoClass comes at a cost: Ghoshal *et al.* (2021) required human experts to manually annotate thousands of immunohistochemical images. Our procedure is much cheaper by comparison, requiring *no human annotation of images*, and is therefore applicable even when expert annotation of cell types at that scale is impractical.

We also believe our practice of downsampling images to 512×512 adversely affected our performance on testis. While cell identity in kidney largely corresponds with macroscopic tissue organization, cells of different types in testis (such as spermatids at different developmental stages) intermix spatially. These therefore likely necessitate finer-scale information (e.g. cellular morphology, chromatin organization) to distinguish (McLachlan *et al.*, 2006), which our downsampling procedure removes. In comparison, DeepHistoClass is trained on full-resolution 3000×3000 images. It is tractable to train that method at full resolution because it is intrinsically less memory intensive, being a supervised learning algorithm with only linear space complexity in batch size. SimCLR is on the other hand quadratic in this requirement (Chen *et al.*, 2020).

References

Chen, T., Kornblith, S., Norouzi, M., and Hinton, G. E. (2020). A simple framework for contrastive learning of visual representations. In *Proceedings of the 37th International Conference on Machine Learning, ICML 2020, 13-18 July 2020, Virtual Event*, volume 119 of *Proceedings of Machine Learning Research*, pages 1597–1607. PMLR.

Ghoshal, B., Hikmet, F., Pineau, C., Tucker, A., and Lindskog, C. (2021). DeepHistoClass: A novel strategy for confident classification of immunohistochemistry images using deep learning. *Molecular & Cellular Proteomics*, **20**, 100140.

Guo, J., Grow, E. J., Mlcochova, H., Maher, G. J., Lindskog, C., Nie, X., Guo, Y., Takei, Y., Yun, J., Cai, L., Kim, R., Carrell, D. T., Goriely, A., Hotaling, J. M., and Cairns, B. R. (2018). The adult human testis transcriptional cell atlas. *Cell Research*, **28**(12), 1141–1157.

McLachlan, R., Meyts, E. R.-D., Hoei-Hansen, C., de Kretser, D., and Skakkebaek, N. (2006). Histological evaluation of the human testis—approaches to optimizing the clinical value of the assessment: Mini review. *Human Reproduction*, **22**(1), 2–16.

Muto, Y., Wilson, P. C., Ledru, N., Wu, H., Dimke, H., Waikar, S. S., and Humphreys, B. D. (2021). Single cell transcriptional and chromatin accessibility profiling redefine cellular heterogeneity in the adult human kidney. *Nature Communications*, **12**(1).
